# Filling-Balance-Oriented Parameters for Multi-Cavity Molds in Polyvinyl Chloride Injection Molding

**DOI:** 10.3390/polym14173483

**Published:** 2022-08-25

**Authors:** Hsi-Hsun Tsai, Shao-Jung Wu, Jia-Wei Liu, Sin-He Chen, Jui-Jung Lin

**Affiliations:** 1 Department of Mechanical Engineering, Ming Chi University of Technology, New Taipei City 24301, Taiwan; 2 Research Center for Intelligent Medical Devices, Ming Chi University of Technology, New Taipei City 24301, Taiwan; 3 Department of Chemical Engineering, Ming Chi University of Technology, New Taipei City 24301, Taiwan; 4 Polymer Department of R&D Center, Nan Ya Plastics Corporation, New Taipei City 23872, Taiwan

**Keywords:** filling balance, imbalance, injection molding, PVC, molding flow analysis, Taguchi method, optimization

## Abstract

PVC injection molding has constrained temperature and shear rate owing to its temperature sensitivity and high viscosity, as well as its low conductivity. Many challenges are associated with the PVC injection molding process used for producing PVC fittings with a multi-cavity mold. Once filling imbalance occurs, the gates and/or runner of the mold should be changed by machine tools, which is time- and cost-intensive. Using Moldex3D and the Taguchi method, this study reveals an approach to eliminate imbalanced filling of multi-cavity molds for PVC injection molding. The injection rate optimization of the filling stage is successfully verified to reduce the imbalance. Furthermore, the temperatures of the molded PVC fittings are only slightly increased by the change in injection rate. The temperatures of fittings in the filling and packing are lower than the degradation temperature of PVC. This approach may help technicians to obtain pilot-run samples for the optimization of molding parameters and ensure degradation-free PVC molding.

## 1. Introduction

Polyvinyl Chloride (PVC) is one of the most widely used polymers due to its versatile nature; it is a durable and long-lasting material used for hundreds of healthcare and construction products around the world. This material accounts for about 20% of all plastic manufactured world-wide, second only to polyethylene [1]. PVC’s flow characteristics lend themselves to extrusion for pipes and continuous parts. Early trials conducted using PVC in injection molding applications met with very unsuccessful results due to the higher viscosity of the melt polymer during the filling stage in plastic injection molding. PVC injection molding meets defects on molded parts which are similar to the other plastics [2]. A different characteristic of PVC injection molding is that the melt PVC is sensitive to temperature, and the thermo-viscoelastic effect gives an additional temperature increase due to this higher shear rate in injection molding [3]. Under a high temperature, melt PVC often decomposes and burns during the injection molding process [4]. Until the low-viscosity PVC compounded for injection molding in the 1980s, injection-molding-grade PVC gradually contributed parts utilized in healthcare, industrial, and consumer goods [5]. High-flow PVC for injection molding has made significant contributions to healthcare and consumer goods, for instance, the fittings of the blood pipelines for hemodialysis in healthcare, transportation pipelines for chemicals in industry, and water pipelines in the consumer market. The fittings of these pipelines directly connect as adaptors and/or veering connect as elbows to the extruded PVC pipes. These fittings are mainly manufactured via a PVC injection molding process. However, the ranges of the melt temperature and filling rate in PVC injection molding are limited due to PVC degradation [6]. Under the real settings of the machine specifications, simulations by molding flow software have been successfully implemented to derive molding parameters for practical operations [7,8,9,10,11]. To avoid PVC degradation during injection molding, simulations may also help to construct a successful set of parameters to enhance the molding efficiency by examining the temperature distribution of the cavity during the filling stage [12]. The taper of the sprue for PVC injection molding may also induce degradation [13].

The complexity of the chemistry of PVC in thermal degradation calls for several lumping procedures to handle it. Many studies have aimed to constrain the degradation of PVC in injection molding. One of the approaches is to add heat stabilizers into the PVC. Traditional added fillers, such as lead and/or calcium zinc, mixed with PVC may increase the temperature of PVC degradation and decrease the release rate of hydrogen chloride (HCl) from PVC. However, the PVC industry made a voluntary commitment to replace lead-based stabilizers by the end of 2015 [14]. Organic-based stabilizer is thus an approach to successfully decrease the HCl release rate from PVC [15]. Another approach is to absorb HCl, which is the start of the degradation chain reaction [16,17]. By using nanometer particles of silica to absorb HCl, it was verified that the degradation temperature was thereby elevated, with a minor effect on color change of the compounded sample [18]. By simulation of the temperature distribution in the filling stage, molding parts can be produced under degradation-free conditions in injection molding. A filling simulation of PVC injection molding must be very particular due to its high viscosity and high temperature sensitivity. The viscosities of the melt PVC in the runners and gates of a mold with multiple cavities are of different levels, even when the runners and gates of the cavities are laid out in a geometric arrangement. These different viscosities in the gates and runners are induced by the non-symmetric distribution of the shear rate, which leads to imbalanced flow fronts into symmetric cavities in the geometry. Two principal factors affect filling imbalance in injection molding: the shear rate distribution of the melt polymer and the temperature distribution of the mold [19]. Simulation is desired to handle this phenomenon of imbalanced filling effectively [20]. Via the three procedures of response surface methodology, the Taguchi method, and artificial neural networks, the filling balance can be optimized on the basis of the injection rate, melt temperature, mold temperature, and geometry of the runner. The artificial neural network approach is the most efficient optimization procedure to solve the imbalance phenomenon [21].

By the Taguchi method and an artificial neural network, a set of optimal process parameters can be derived. The most important factor of the injection parameters affecting filling imbalance is the shear rate of the melt polymer [22]. A “melt flipper”, which is a melt rotation technology, was inserted at the intersection of the primary and secondary runners to reduce the shear rate variation across the melt [23,24]. The numerical results showed that this flipper reduced the imbalance in an “H”-patterned eight-cavity runner system [25]. Geometrically balanced multi-cavities of a mold do not ensure uniform filling in injection molding. Under a greater filling pressure, the firstly filled cavity may induce leakage of the melt polymer. Polymer leakage occurs in the form of plastic burrs associated with the molded parts acquired under this filling imbalance in plastic injection molding. Filling imbalance can also lead the firstly filled cavity to an over-packing situation [7]. The short-shot technique by an empirical approach [10,11] can reveal filling imbalance. Based on the revealed results, the sizes of the runners and/or gates of the mold can be changed using machine tools via a traditional trial-and-error approach. In the design phase, simulation is an essential approach to define the sizes of the runners and gates of a multi-cavity mold.

Before injection molding, molding flow simulations, in the early stage of the design phase, reveal not only the temperature distribution under degradation of PVC injection molding, but also the filling imbalance of multiple cavities of the mold. Using the simulation results, the design details on the mold dimensions can be changed to optimize the design, and the operation parameters for real trials can be enhanced for successful molding. Once filling imbalance is present within the injection mold, the mold must be dismantled to change the sizes of runners and/or gates using machine tools, which increases the cost of time and tooling. More efforts are required to address filling imbalance in PVC injection molding thanks to its temperature sensitivity affecting the viscosity of the melt polymer. Beyond changes to the sizes of runners and gates, this study aims at constructing a multi-stage injection rate method in the filling stage using Moldex3D [26] to reduce the filling imbalance of a multi-cavity mold for PVC injection molding. Based on existing imbalanced PVC fittings, the numerical model under the PVC properties, injection parameters, and machine is compared. Using the Taguchi method to optimize the molding parameters, a new setting for a multi-stage injection rate is established and implemented to reduce filling imbalance in the multi-cavity mold. With this new injection rate setting, experimental injection-molded PVC fittings are then used to verify the numerical results.

## 2. Materials and Methods

**Figure 1:**
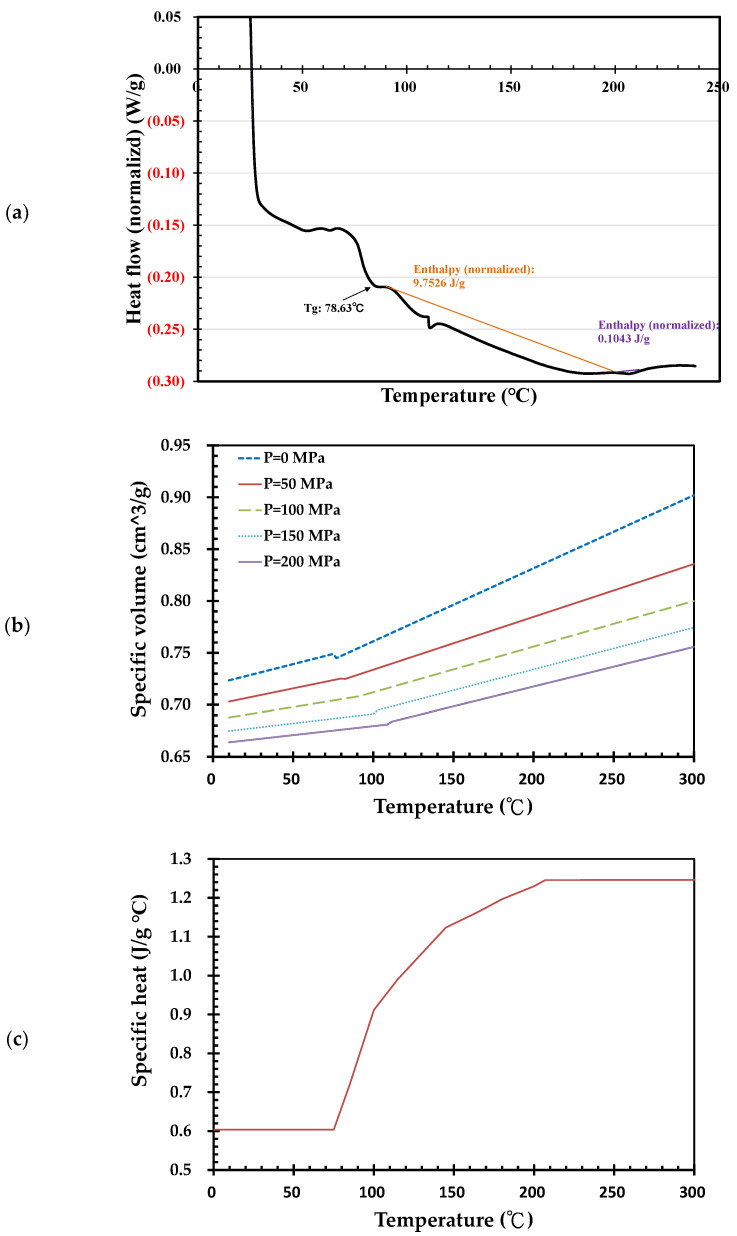

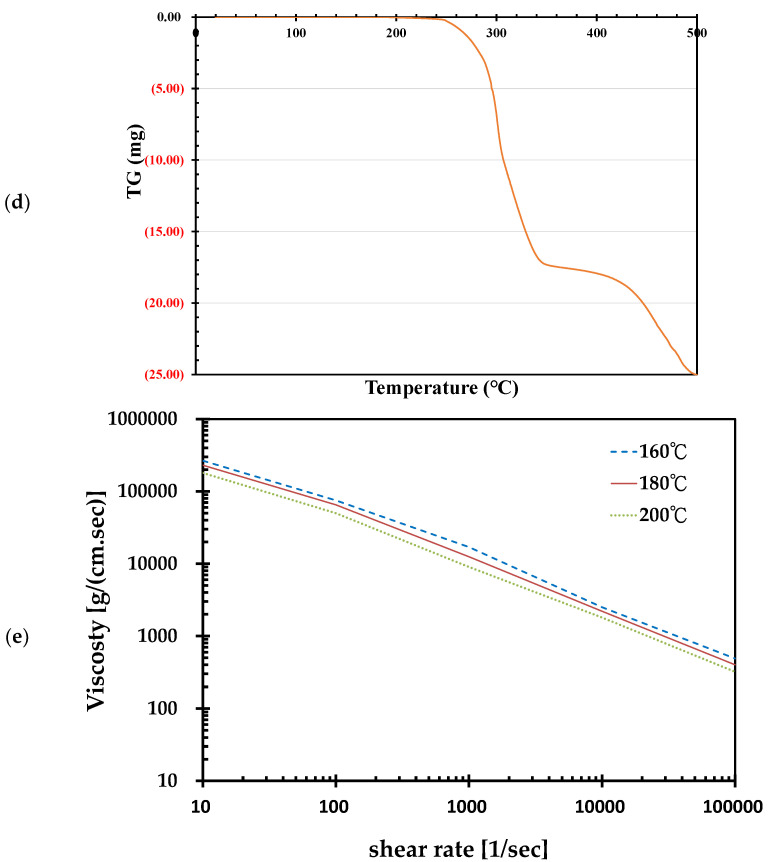
Rheological properties of Suspension S-60 PVC, Formosa Plastic Co. (**a**) DSC heating curves; (**b**) P-v-T curves; (**c**) specific heat curve; (**d**) thermo-gravimetric analysis results; (**e**) viscosity.

The polymer in this study is a rigid PVC of Suspension S-60 [27], from Formosa Plastic co. Suspension S-60 is a standard material used for the injection molding of PVC fittings [28]. The following are some of the properties used for the simulation: density, 1400 kg/m^3^; coefficient of thermal conductivity, 0.08 W/(m °C); and Vicat softening Temperature, 76 °C. The PVC was measured using a differential scanning calorimetry (DSC) instrument (TA Instruments Discovery DSC 25, New Castle, DE, USA) under a nitrogen atmosphere. Suspension S-60 PVC was heated to 240 °C under a ramp of 10 °C/min, held isothermally for 1 min, cooled to 25 °C at a rate of 10 °C/min, held isothermally for 1 min, and heated to 250 °C again at 10 °C/min for the melting temperature measurements. As shown in Figure 1a, the Suspension S-60 PVC analyzed via DSC presented starting melt temperatures of about 109.9 °C; a peak melting temperature occurred at 111.1 °C. The heating enthalpies of the first and second peaks were 9.7526 J/g and 0.1043 J/g, respectively. The glass transition temperature was 74.9 °C. Figure 1b depicts the PvT data (Pressure, Volume, Temperature) of Suspension S-60 PVC measured using a pvT-500, GÖTTFERT (Buchen, Germany). The specific volume of Suspension S-60 PVC varies with pressure and temperature. Under zero pressure, the specific volume can change significantly at the 77 °C encountered during processes such as the injection molding of PVC. The significant changes in specific volume in this figure gradually increase with respect to the endured pressure. In Figure 1c, the specific heat curve of Suspension S-60 PVC was also measured using the previously mentioned DSC instrument. Below 75 °C, the specific heat of PVC is constant. The specific heat increases when the temperature is greater than 75 °C. The maximum value of the specific heat of PVC occurred at 207 °C. Thermo-gravimetric analysis was applied to derive the degradation temperature of PVC using a Netzsch STA 409PC. Suspension S-60 PVC showed weight loss at 210 °C in Figure 1d. Careful control of the PVC temperature below this temperature may avoid thermal degradation of the PVC. Figure 1e shows that the shear rate and temperature of the S-60 PVC are negatively proportional to their viscosity.

Below 110 °C, the thermal conductivity of PVC is 6.254 × 10^−4^ (W/cm °C), while from 110 to 170 °C, the function of thermal conductivity of melt PVC is 5.698 + 0.005055T (W/cm °C). The thermal conductivity of melt PVC is 8.024 × 10^−4^ (W/cm °C) once the temperature is greater than 190 °C. The viscosity of this Suspension S-60 PVC is given by the modified Cross model [26] as

(1) $$\eta \left(\frac{g}{cm\;sec}\right)=\eta_{0}/\left[1+{\left(\frac{\eta_{0}\dot{\gamma }}{\tau^{*}}\right)}^{\left(1-n\right)}\right]$$

where

 η0gcm sec=1.17×1011exp−17.9709T−346.74T−295.14

$\dot{\gamma }$ is the shear rate of molten PVC, *n* = 0.257813,$\;\tau^{*}\left(\frac{N}{cm^{2}}\right)=28.2$, and *T* is the temperature of the molten PVC in degrees Kelvin.

**Figure 2:**
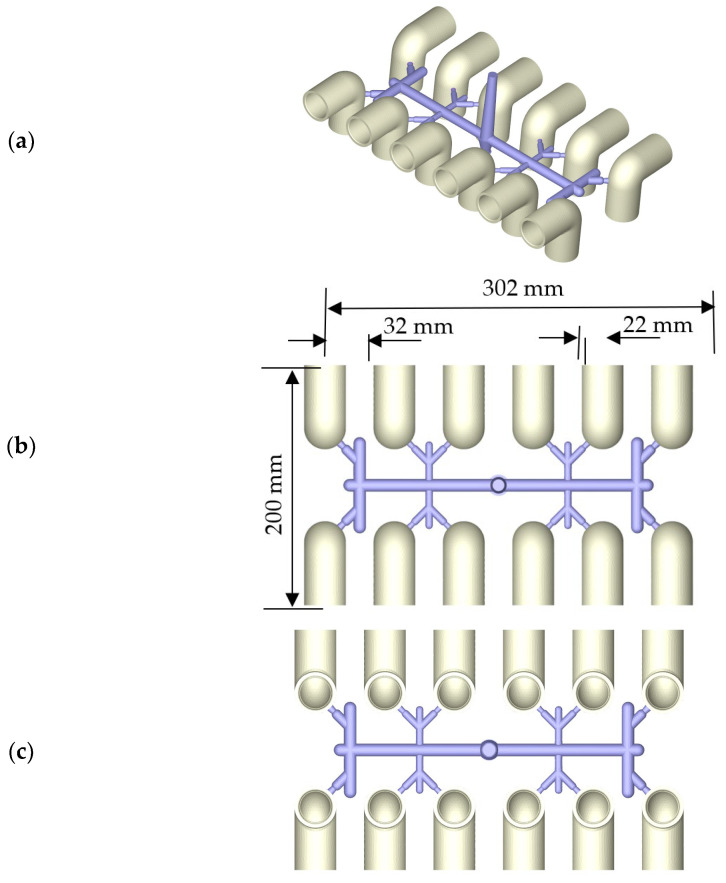

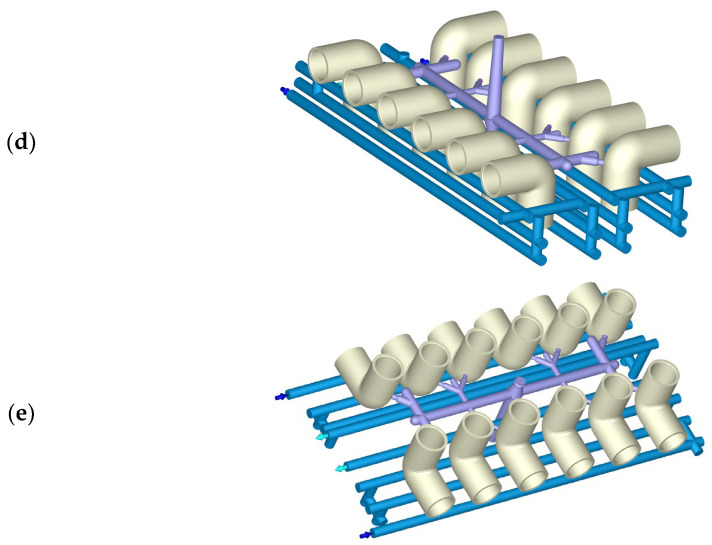
Injection-molded PVC elbows: (**a**) isometric view of the twelve fittings and runners; (**b**) top view of the twelve fittings and runners (302 mm × 200 mm × 60 mm); (**c**) bottom view of the twelve fittings and runners; (**d**) isometric view of the cooling channel within the core plate; (**e**) isometric view of the cooling channel within the cavity plate.

Figure 2 shows the molding parts of twelve PVC fittings. Each fitting has a 32 mm outer diameter and a 90° elbow for water supply. The thickness of this fitting is 3.5 mm. The diameter of primary runners is 10 mm, and the secondary runners are 8 mm in diameter for the outer four cavities and 7 mm in diameter for the others. The filling gates of the twelve cavities are 3.0–4.2 mm. Figure 2a shows an isometric view of the molded parts including the runners and the core of the sprue. Figure 2b shows the top side of the molded 90° elbows. The whole sizes of the molded parts are 302 mm in length, 200 mm in width, and 60 mm in height. The pitch of the cavity is 22 mm. The bottom side of the molded 90° elbows is shown in Figure 2c. Figure 2d presents an isometric view of cooling channels within the core plate and molded parts, as well as the runner. Straight cooling pipes compose the cooling channels around the molded parts. The cooling channel within the cavity plate is shown in Figure 2e.

The filling gates 4.2–5.0 mm in diameter allow the high-viscosity molten PVC material to flow into the mold cavity. The diameter of the primary runner is 9.8 mm, and those of the secondary runner are 8.0 mm for the outer four cavities and 6.0 mm for the inner eight cavities. Each fitting elbow of a cavity is 32.7 cm^3^ in volume. The gates and runner have a volume of 46.2 cm^3^. The solid mesh of the molded parts has 268,440 elements. The mesh of the cold runner has 36,814 elements. As shown in Table 1, the initial injection parameters were a mold temperature of 40 °C, a PVC melt temperature of 175 °C, a filling time of 12.197 s, a cooling time of 40 s, a mold opening time of 5 s, and a cycle time of injection molding of 57.8 s.

Within the experiment and simulation, a 260-ton CI-260E injection machine (Creator, Kaohsiung, Taiwan) was used. This molding machine has screw diameters of 66 mm in the feed section, 85 mm in the transition section, and 77 mm in the melting section; a maximum screw stroke of 225 mm; a maximum injection pressure of 173.4 MPa; and a maximum injection weight of 626 g. The revolution speed of the screw is 25 rpm. The simulation analysis was performed using Moldex3D software. Following the pvT model in Figure 1b, the melt PVC is a compressible and generalized non-Newtonian fluid under the modified Cross model in Equation (1). In the filling phase, the velocity and temperature are specified at the mold inlet. On the mold wall, the non-slip boundary condition is applied, and a fixed mold wall temperature is assumed. The finite volume method was used to discretize the Navier–Stokes equation based on the pressure-based decoupled procedure and solve the transient flow field in a complex three-dimensional geometry in Moldex3D. Modeling the flow field in Moldex3D is an iterative decoupled procedure for coupling velocity and pressure, in which the linearized momentum equations are solved for an initial estimated pressure field, followed by the solution of the pressure correction equation. Then, the mass fluxes and pressure are corrected in iterative calculations until the prescribed convergence condition [9].

**Figure 3:**
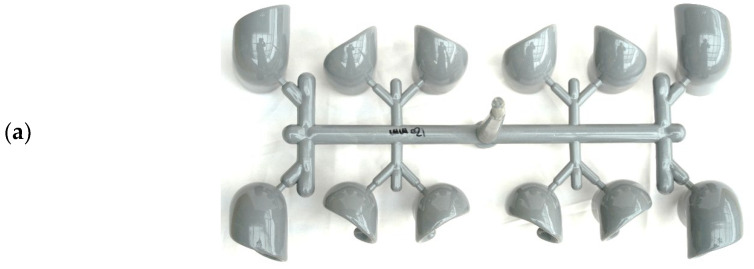

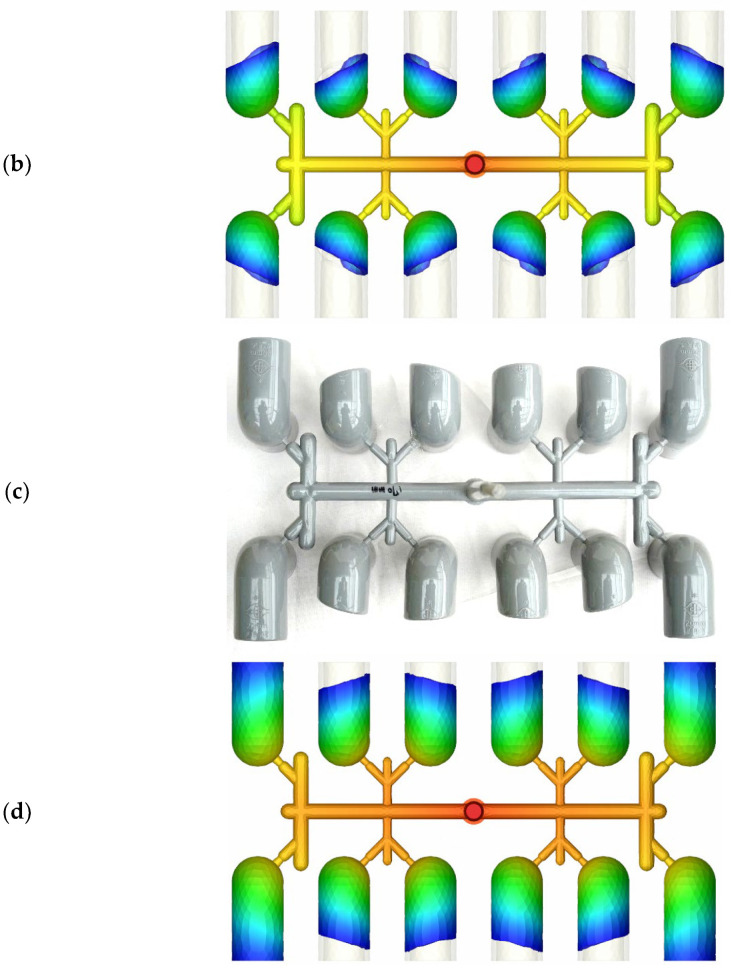
Top views of experimental and numerical comparisons of PVC fittings and runners by short-shot testing: (**a**) experimentally 75% filled parts; (**b**) numerically 75% filled flow front; (**c**) experimental imbalanced-fill parts; (**d**) numerical flow front of imbalanced-fill parts.

According to the injection parameters and machine, the molded parts under 75% and 98% filling by short-shot testing are presented in Figure 3. A 75% short-shot sample was produced by the injection machine, shown in Figure 3a. This 75% short-shot of the experimental injection PVC fitting was compared to the flow front on the top side of the PVC molded parts via a numerical approach, as shown in Figure 3b. The profile of the experimental PVC molded parts agrees qualitatively with the simulated flow front of the parts. In Figure 3c, the profile of the top surface of the PVC molded fittings produced by about 98% short-shot testing indicates a close fit with the numerical flow front, as shown in Figure 3d. The outer four cavities of the mold are totally filled with melt PVC. However, the inner eight cavities are short-filled with melt PVC. This filling imbalance in the multi-cavity mold provides significant evidence of the prediction accuracy of the experimental and numerical results.

For improvement of the filling imbalance, a traditional approach is to enlarge the sizes of the gates and secondary runners of the mold to better balance the filling. In this study, the injection rates of four stages during filling were set at several levels according to the Taguchi experimental plan to reducing filling imbalance beyond the enlargements of gates and runners. The optimized four stages of filling rates at three different levels are selected and tabulated in Table 2. The interactions between the parameters were not considered in this study. The levels of the parameters were selected based on the empirical operations and discussions through the injection molding process. From the number of parameters and number of levels in Table 3, the use of the experimental layout L9 model was carried out to derive the responses by smaller-is-better signal to noise (S/N ratio) estimation. The effects induced by the noise factor, the uncontrolled factor in system, should be minimized since the noise is the result from all errors encountered in experiment. A higher value of S/N ratio indicates a minimum effect of the noise factor [29,30]. By S/N ratio, Taguchi method identifies the control factors and then moves the mean to target a smaller effect of the S/N ratio for optimization of the levels of control factors. In this study, the smaller differential imbalance fillings indicate better values, the corresponding S/N ratio (dB) is

−10log10∑i=1nyin

where *n* is the number of replications and *y* is the experimental value [31].

## 3. Results and Discussion

### 3.1. Time Delay of an Imbalance in Filling

**Figure 4:**
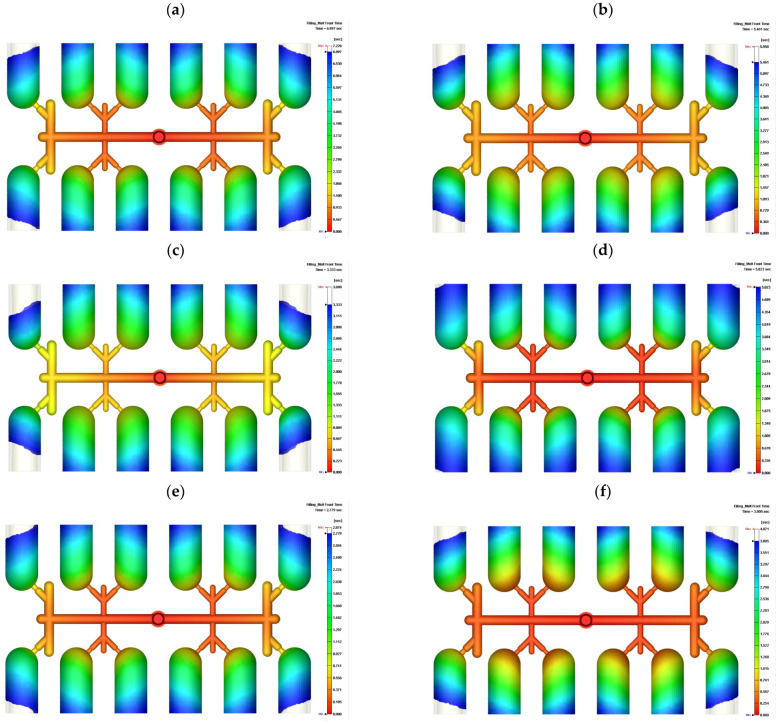

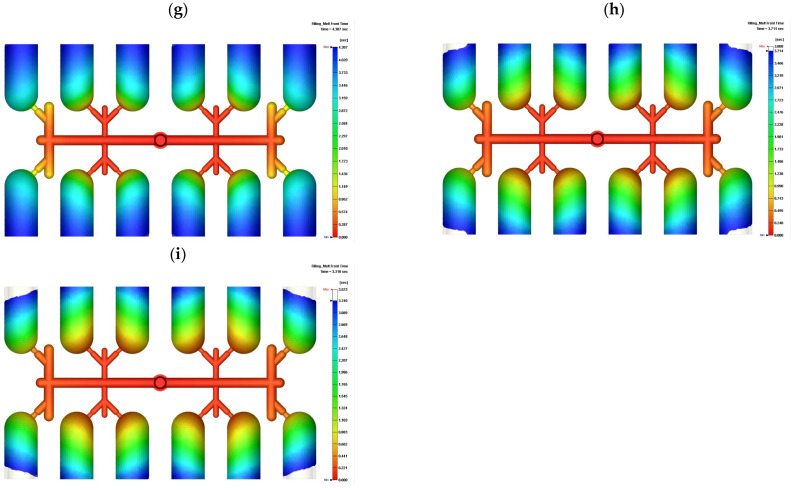
Top view of the filling time and time delay of imbalance by Moldex3D simulation following the L9 orthogonal array: (**a**) Trial 1, filling time 7.228 s; (**b**) Trial 2, filling time 5.958 s; (**c**) Trial 3, filling time 3.69 s; (**d**) Trial 4, filling time 5.066 s; (**e**) Trial 5, filling time 2.874 s; (**f**) Trial 6, filling time 4.071 s; (**g**) Trial 7, filling time 4.319 s; (**h**) Trial 8, filling time 3.808 s; (**i**) Trial 9, filling time 3.523 s.

In the trial simulation following the plan in Table 3, the time of the filling front in the Trial 1 simulation was 7.228 s and the time delay of filling was 0.195 s, as shown in Figure 4a. The delay time of filling means the time difference between the final filled front and the first filled front. The molten PVC spread to all twelve cavities within 7.228 s under 10%, 10%, 30%, and 30% injection rates at the first to fourth stages of filling, respectively. The ratio of time delay to filling time is noted as the percentage of filling time delay of imbalance, at 2.77%. Across all nine simulation trials, the maximum filling temperature, filling time, time delay of imbalance, and percentage of filling time delay of imbalance are listed in Table 4.

### 3.2. Optimization via the Taguchi Method and Verification

In manufacturing process, Taguchi method has been widely employed to optimize the process parameters for output quality [32]. Based on the literature review, the control parameters are chosen through the injection process for the evaluation of the selected temperature increases. Additionally, based on the number of parameters and number of levels, an L9 orthogonal array is selected, as shown in Table 4. To reduce multi-cavity filling imbalance, the “the smaller the better” quality characteristic was chosen [21]. The signal-to-noise ratios were calculated and tabulated in Table 5. The next step was to determine the Taguchi response table to find the most significant parameters from the selected parameters and their optimum levels, shown in Table 6. The optimum level is, hence, the highest value of each parameter. In Table 6, the best level of each injection rate is indicated by the bottom line. To reduce the imbalance time delay, we should set the 1st stage injection rate at level 3, the 2nd stage injection rate at level 1, the 3rd stage injection rate at level 3, and the 4th stage injection rate at level 3.

**Figure 5:**
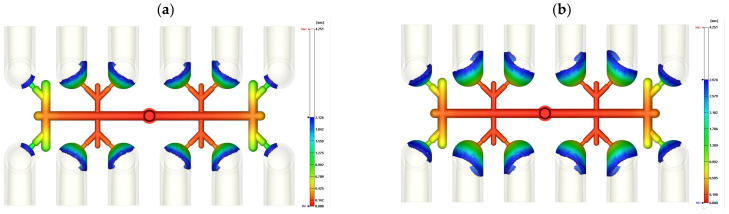

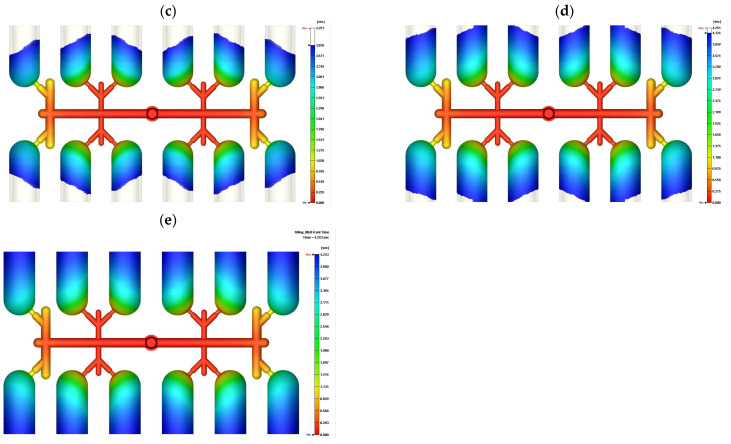
Flow fronts of different filling percentages under optimization of the injection rate to achieve balanced filling of a multi-cavity mold: (**a**) 50% filling; (**b**) 70% filling; (**c**) 90% filling; (**d**) 97% filling; (**e**) time delay 0.008 s.

Simulation verification is an essential procedure to derive the optimization result based on the optimal levels of parameters. The filling fronts at 50%, 70%, 90%, and 99.6% are shown in Figure 5. The inner eight cavities show greater spreading of the fronts at 50% and 70% filling. The fronts of the outer four cavities catch up to this spread, such that the fronts of all cavities are at nearly the same spread in Figure 5c,d. The time delay of these optimized injection rates is 0.008 s, which is a percentage of 0.19%. This time delay and percentage with the optimized parameters are smaller than those in Trial 7 of the L9 orthogonal array. By the Taguchi method, the filling imbalance is successfully reduced to a near-zero value. This study thus shows an approach to eliminate imbalance in multi-cavity filling beyond the enlargement of gates and runners using machine tools.

### 3.3. Temperature, Shear Rate and Pressure Distributions

**Figure 6:**
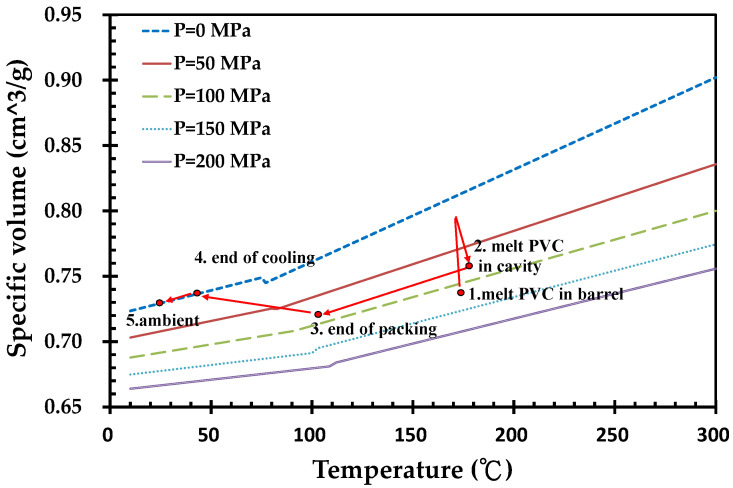
Temperature, pressure and specific volume of PVC during injection molding process.

The shear rate of melt PVC during the filling stage may enhance the temperature of the molten PVC. Figure 1d indicates that the weight loss increases at 210 °C. The temperature distribution of the molding parts during the filling and packing stages was therefore fundamentally examined. In Figure 6, the melt PVC in barrel of injection machine has 175 °C in temperature and 112.7 MPa in injection pressure at point 1. At the end of filling stage, the average and maximum temperatures of parts in cavity are 109.8 °C and 188.3 °C, respectively, meanwhile, the average and maximum pressures of parts are 10.1 and 55.3 MPa, respectively. Then, the parts under the packing pressure of 121.4 MPa are cooled until the end of packing. At point 3, the average and maximum temperatures of parts are 96.4 °C and 177.0 °C, respectively. The molded parts are then cooled down to 47.8 °C in average temperature and 135.8 °C in maximum temperature at the end of cooling stage. The molded parts are then ejected from the mold and cooled down to ambient temperature at point 5 of the figure.

**Figure 7:**
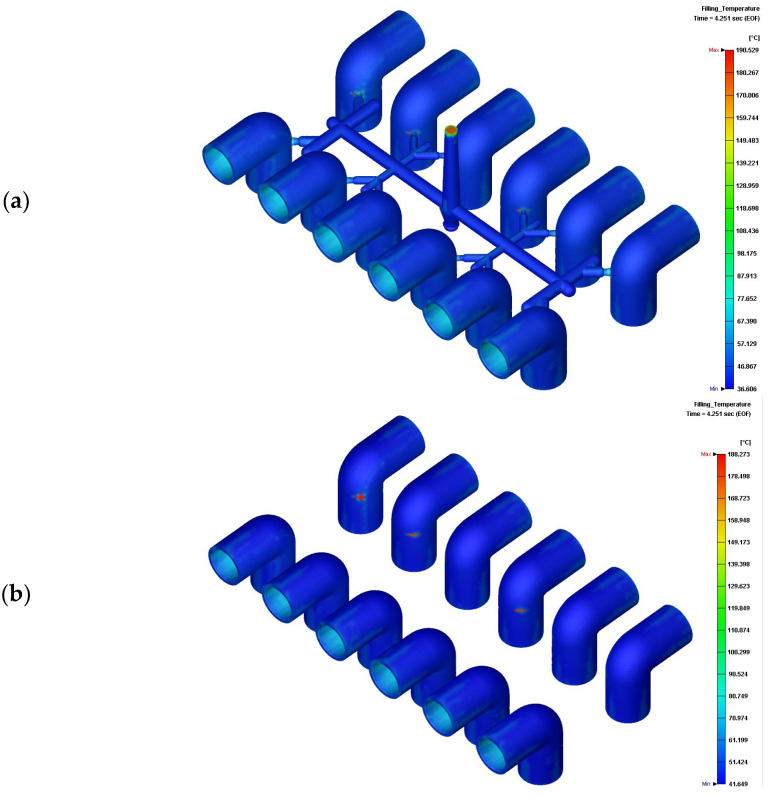

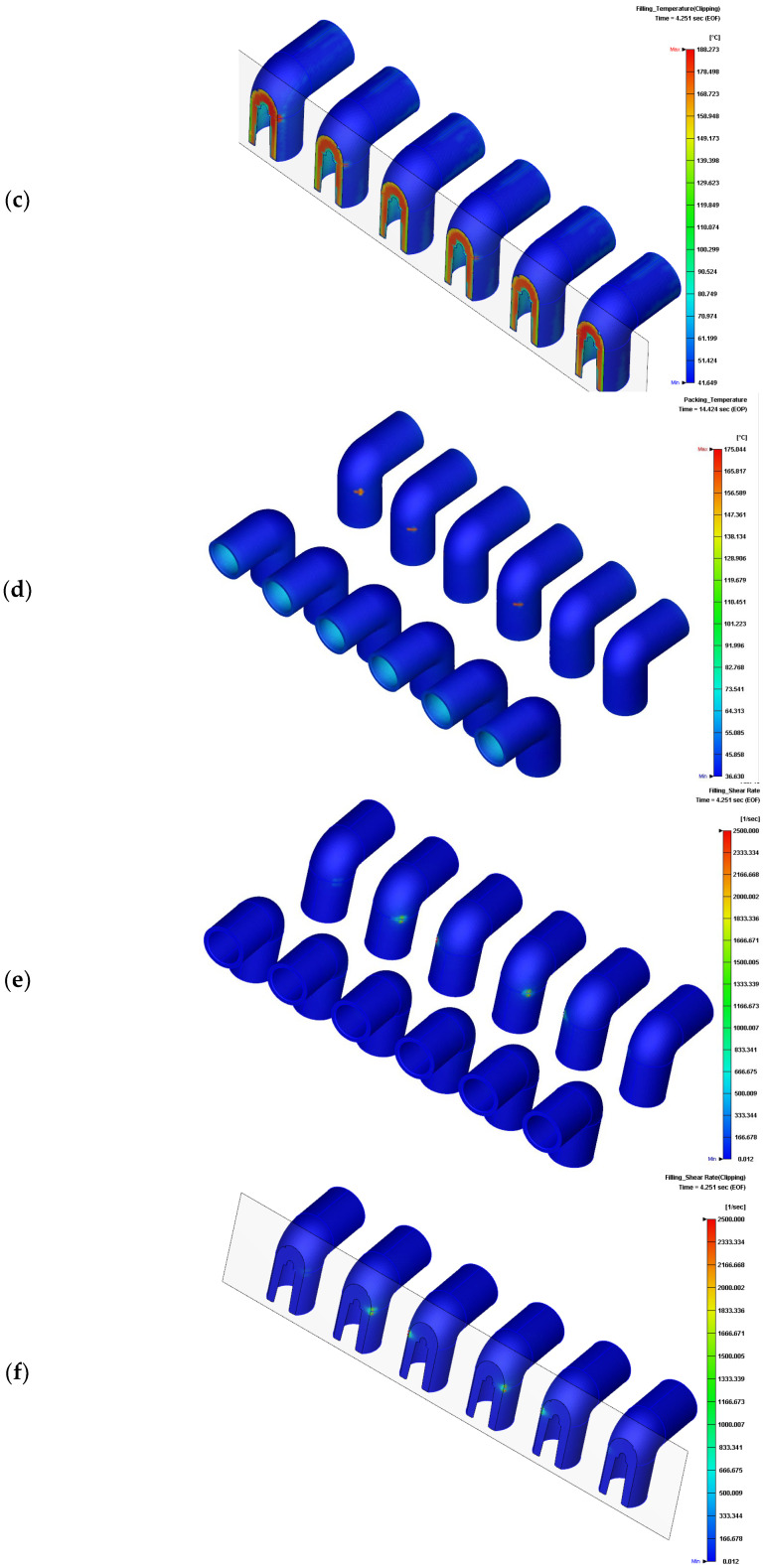

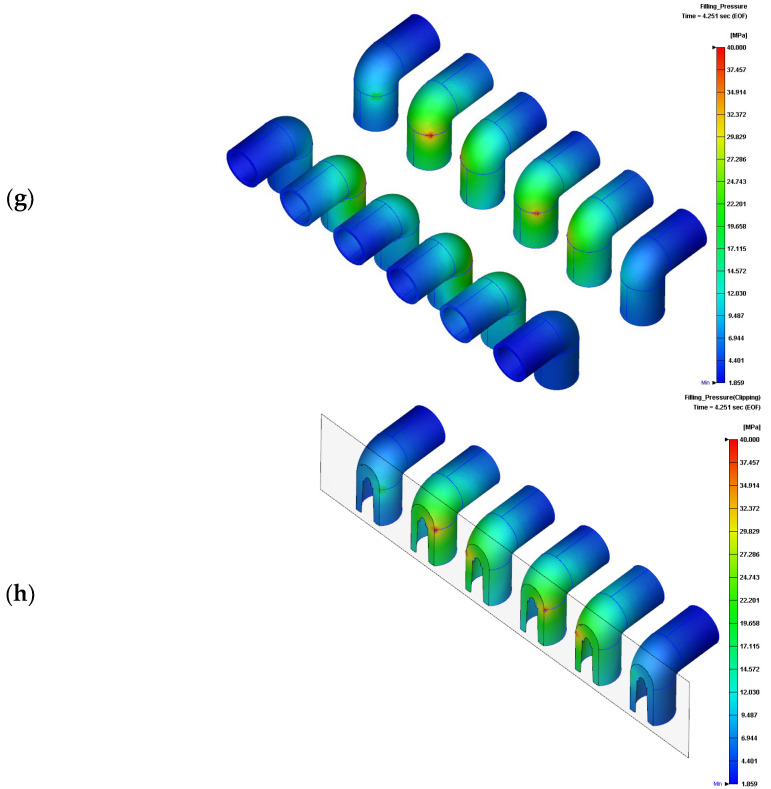
Temperature and pressure distributions of molded parts: (**a**) temperatures of the runners and parts at the end of filling stage; (**b**) temperature of parts at the end of filling stage ; (**c**) clipping view of the temperature distribution of parts at the end of filling stage; (**d**) temperature of parts in the packing stage; (**e**) shear rate distribution during filling stage; (**f**) clipping view of the maximum shear rate during filling stage; (**g**) filling pressure distribution of molded parts; (**h**) clipping view of the filling pressure distribution of molded parts.

Figure 7a shows that the maximum temperature of the parts and runners is 190.5 °C. If we exclude the runners, the parts show a maximum temperature of 180.3 °C on the gates and within the parts in the filling stage (Figure 7b,c) and a maximum temperature of 175.0 °C in the packing stage (Figure 7d). The shear rates of melt PVC on gates are much higher than the others, the temperature of gates are thus enhanced by the shear rates [33]. These temperatures are lower than the degradation temperature. Optimization of the injection rate would thus successfully eliminate filling imbalance and ensure degradation-free molten PVC in the filling and packing stages of injection molding. The shear rate may decrease the viscosity of melt PVC, however, in Figure 7e the maximum shear rate is 2800 (1/s). For detailed examination the distribution of shear rate in clipping view of the molded parts depicted in Figure 7f, the maximum shear rate occurred in the gates. The average shear rate of molded parts in cavity during filling stage is 8.9 (1/s). By the maximum and average shear rates, the viscosity respect to the shear rate and temperature in Figure 1d indicates the viscosity of melt PVC is from 100,000 to 190,000 (g/cm s) which is much higher than the ones of general polymers in injection molding. Figure 7g,h dedicate 40 MPa in the maximum pressure of PVC parts at the end of filling stage although the injection pressure is 112.7 MPa. The gradient of pressure of melt PVC is high due to its high viscosity.

## 4. Conclusions

Beyond making changes to the sizes of runners and gates of a mold using machine tools once a filling imbalance occurs, this study presented an approach to set a multi-stage injection rate in the filling process by way of Moldex3D and the Taguchi method. The verified results show that the filling imbalance in the multi-cavity mold for PVC injection molding was successfully eliminated. Besides this, the temperatures of the molded parts were only slightly increased by the injection rate. The temperatures of the parts in the filling and packing stages were far from the degradation temperature. This approach may help technicians to obtain pilot-run samples to optimize molding parameters and ensure degradation-free PVC molding.

## Figures and Tables

**Table 1:** Initial injection molding parameters of Suspension S-60 PVC for fittings.

Parameter	Value
Mold temperature (°C)	40
Filling pressure (max) (MPa)	173.4
Filling rate (max) (mm^3^/s)	376,000
Melt temperature (°C)	175
Filling time (s)	12.197
Filling rate of 1st stage (ram 227.7–217.6 mm) (%)	16
Filling rate of 2nd stage (ram 217.6–175.5 mm) (%)	7.6
Filling rate of 3rd stage (ram 175.5–131.4 mm) (%)	12.5
Filling rate of 4th stage (ram 131.4–0 mm) (%)	15
V/P switch (at ram) (mm)	12
Filling volume (mm^3^)	438,600
Packing time (s)	10
Packing pressure (MPa)	121.38
Cooling time (s)	40
Cooling channel temperature (°C)	30
Mold opening time (s)	5
Cycle time (s)	57.8

**Table 2:** Injection molding parameters of Suspension S-60 PVC.

Parameter	Level 1	Level 2	Level 3
1st stage injection rate (%)	10	30	50
2nd stage injection rate (%)	10	25	40
3rd stage injection rate (%)	30	50	70
4th stage injection rate (%)	30	50	70

**Table 3:** L9 orthogonal array.

Trial Number	1st Stage Injection Rate Level	2nd Stage Injection Rate Level	3rd Stage Injection Rate Level	4th Stage Injection Rate Level
1	1	1	1	1
2	1	2	2	2
3	1	3	3	3
4	2	1	2	3
5	2	2	3	1
6	2	3	1	2
7	3	1	3	2
8	3	2	1	3
9	3	3	2	1

**Table 4:** Defect determination in the 9 experiments based on Moldex3D simulation results.

Trial Number	Max. Filling Temperature (°C)	Filling Time (s)	Filling Time Delay of Imbalance (s)	Percentage of Filling Time Delay of Imbalance (%)
1	183.25	7.228	0.231	3.30
2	185.86	5.958	0.497	9.10
3	183.14	3.690	0.357	10.70
4	187.26	5.044	0.021	0.42
5	184.94	2.874	0.095	3.42
6	185.35	4.071	0.266	6.99
7	188.40	4.319	0.012	0.28
8	187.55	3.808	0.094	2.53
9	186.04	3.523	0.213	6.44

**Table 5:** Signal-to-noise ratios for “the smaller the better” quality characteristics.

Trial Number	1st Stage Injection Rate Level	2nd Stage Injection Rate Level	3rd Stage Injection Rate Level	4th Stage Injection Rate Level	S/N
1	1	1	1	1	−10.37
2	1	2	2	2	−19.18
3	1	3	3	3	−20.60
4	2	1	2	3	7.57
5	2	2	3	1	−10.68
6	2	3	1	2	−16.89
7	3	1	3	2	−11.10
8	3	2	1	3	−8.07
9	3	3	2	1	−16.17

**Table 6:** Taguchi response table.

Level	1st Stage Injection Rate Level	2nd Stage Injection Rate Level	3rd Stage Injection Rate Level	4th Stage Injection Rate Level
L1	−15.86	0.83	−9.74	−11.72
L2	−8.35	−11.38	−11.41	−8.16
L3	−3.42	−17.08	−6.48	−7.75
Difference	12.44	16.25	5.03	3.97

## Data Availability

All the data generated or analyzed during this study are included in the published article.

## References

[1] Percentage of PVC among Plastics. https://www.bpf.co.uk/plastipedia/polymers/PVC.aspx.

[2] Ahmed T., Sharma P., Karmaker C.L., Nasir S. (2022). Warpage prediction of Injection-molded PVC part using ensemble machine learning algorithm. Mater. Today Proc..

[3] Koszkul J., Nabialek J. (2004). Viscosity models in simulation of the filling stage of the injection moulded process. J. Mater. Process. Technol..

[4] Garcia J.L., Koelling K.W., Xu G., Summers J.W. (2004). PVC degradation during injection molding: Experimental evaluation. J. Vinyl Technol..

[5] Garcia J.L., Koelling K.W., Summers J.W. (2004). Computational prediction of PVC degradation during injection molding in a rectangular channel. Polym. Eng. Sci..

[6] Weir S. (1994). Predicting surface defects in injection molded PVC components. J. Vinyl Technol..

[7] Yavari R., Khorsand H. (2021). Numerical and experimental study of injection step, separation, and imbalance filling in low pressure injection molding of ceramic components. J. Eur. Ceram. Soc..

[8] Fernandes C., Pontes A.J., Viana J.C., Gaspar-Cunha A. (2016). Modeling and Optimization of the Injection-Molding Process: A Review. Adv. Polym. Technol..

[9] Chang R.-Y., Yang W.-H. (2001). Numerical simulation of mold filling in injection molding using a three-dimensional finite volume approach. Int. J. Numer. Methods Fluids.

[10] Tsai H.H., Liao Y.L. (2022). Feasibility Study of the Flatness of a Plastic Injection Molded Pallet by a Newly Proposed Sequential Valve Gate System. Polymers.

[11] Liao Y.-L., Tsai H.-H. (2022). A Comparison of Numerical and Actual Measurements of Large-Scale Rib-Structured Pallet Flatness Using Recycled Polypropylene in Injection Molding. Polymers.

[12] Lladó J., Sánchez B. (2008). Influence of injection parameters on the formation of blush in injection moulding of PVC. J. Mater. Processing Technol..

[13] Sánchez B., Lladó J. (2008). Surface quality of PVC fittings based on the design of the sprue. J. Mater. Processing Technol..

[14] Impact of Lead Restrictions on the Recycling of PVC. https://www.vinylplus.eu/resources/impact-of-lead-restrictions-on-the-recycling-of-pvc/.

[15] Jubsilp C., Asawakosinchai A., Mora P., Saramas D., Rimdusit S. (2022). Effects of Organic Based Heat Stabilizer on Properties of Polyvinyl Chloride for Pipe Applications: A Comparative Study with Pb and CaZn Systems. Polymers.

[16] Yu J., Sun L.S., Ma C., Qiao Y., Yao H. (2016). Thermal degradation of PVC: A review. Waste Manag..

[17] Xu Z., Kolapkar S.S., Zinchik S., Bar-Ziv E., Mcdonald A.G. (2020). Comprehensive kinetic study of thermal degradation of polyvinylchloride (PVC). Polym. Degrad. Stab..

[18] Tomaszewska J., Sterzyński T., Walczak D. (2021). Thermal Stability of Nanosilica-Modified Poly(vinyl chloride). Polymers.

[19] Wilczyński K., Narowski P. (2018). Experimental and theoretical study on filling imbalance in geometrically balanced injection molds. Polym. Engr. Sci..

[20] Wilczyński K., Narowski P. (2019). Simulation studies on the effect of material characteristics and runners layout geometry on the filling imbalance in geometrically balanced injection molds. Polymers.

[21] Wilczyński K., Narowski P. (2020). A Strategy for Problem Solving of Filling Imbalance in Geometrically Balanced Injection Molds. Polymers.

[22] Moayyedian M., Dinc A., Mamedov A. (2021). Optimization of Injection-Molding Process for Thin-Walled Polypropylene Part Using Artificial Neural Network and Taguchi Techniques. Polymers.

[23] Narowski P., Wilczyński K. (2015). Study on filling patterns of engineering polymers in geometrically balanced injection molds. Chall. Mod. Technol..

[24] Beaumont J.P., Young J.H., Jaworski M.J. (1999). Mold Filling Imbalances in Geometrically Balanced Runner Systems. J. Reinf. Plast. Compos..

[25] Beaumont J.P., Young J.H., Jaworski M.J. (1998). Solving mold filling imbalances in multi-cavity injection molds. J. Inject. Molding Technol..

[26] Moldex3D 2020. https://www.moldex3d.com/.

[27] Formosa Plastic PVC Blend. http://www.fpc.com.tw/fpcw/index.php?op=proL&f=1&s=6.

[28] Nan-Ya PVC Fitting. https://www.npc.com.tw/j2npc/enus/proddoc/Pipes%20&%20Fittings/PVC-U%20Pipes%20and%20Fitting%20catalog?type=info&docid=F00000177enus1&pdid=F00000177.

[29] Gaaz T.S., Sulong A.B., Kadhum A.A.H., Nassir M.H., Al-Amiery A.A. (2016). Optimizing Injection Molding Parameters of Different Halloysites Type-Reinforced Thermoplastic Polyurethane Nanocomposites via Taguchi Complemented with ANOVA. Materials.

[30] Mehat N.M., Kamaruddin S. (2012). Quality control and design optimisation of plastic product using Taguchi method: A comprehensive review. Int. J. Plast. Technol..

[31] Ryu Y., Sohn J.S., Kweon B.C., Cha S.W. (2019). Shrinkage Optimization in Talc- and Glass-Fiber-Reinforced Polypropylene Composites. Materials.

[32] Tang S.H., Tan Y.J., Sapuan S.M., Sulaiman S., Ismail N., Samin R. (2007). The use of Taguchi method in the design of plastic injection mould for reducing warpage. J. Mater. Proc. Technol..

[33] Marra F., Liparoti S., Speranza V., Pantani R. (2022). Morphology predictions in molded parts: A multiphysics approach. Chem. Eng. Res. Des..

